# Successful Treatment of High-Level Aminoglycoside-Resistant *Enterococcus faecalis* Bacteremia in a Preterm Infant with Ampicillin and Cefotaxime

**DOI:** 10.1155/2018/7567914

**Published:** 2018-03-18

**Authors:** Jennifer Tam, Santina J. Lee, Vibhuti Shah, Shaun K. Morris

**Affiliations:** ^1^Division of Infectious Diseases, The Hospital for Sick Children, 555 University Ave., Toronto, ON, Canada; ^2^Department of Pediatrics, Faculty of Medicine, University of Toronto, 1 King's College Circle, Room 2109, Toronto, ON, Canada; ^3^Department of Pediatrics and Child Health, University of Manitoba, 840 Sherbrook St., Winnipeg, MB, Canada; ^4^Department of Paediatrics, Mount Sinai Hospital, 600 University Ave., Toronto, ON, Canada; ^5^Institute of Health Policy, Management and Evaluation, University of Toronto, 155 College Street, Suite 425, Toronto, ON, Canada

## Abstract

Enterococcal bloodstream infections are usually treated with single-agent antibiotics. In persistent infections, synergistic combination therapy is often required with a beta-lactam and an aminoglycoside antibiotic. High-level aminoglycoside-resistant (HLAR) enterococci are increasingly prevalent and preclude the use of this combination. The use of ampicillin with a third-generation cephalosporin to treat endovascular HLAR *Enterococcus* infections is becoming more established in the adult population; however, the literature on treatment of such infections in children remains scarce. We report a preterm neonate with persistent HLAR *Enterococcus faecalis* bacteremia from day of life 9 to 17 despite treatment with ampicillin and vancomycin. On day of life 17, antibiotic treatment was switched to ampicillin and cefotaxime, with subsequent clearance of blood cultures on day of life 20. To our knowledge, this is the first report illustrating the use of ampicillin and cefotaxime for an HLAR *E. faecalis* infection in a neonate.

## 1. Introduction

Uncomplicated enterococcal bloodstream infections are usually treated with single-agent antibiotics. Persistent infections, however, often warrant treatment with a synergistic combination of a beta-lactam and an aminoglycoside antibiotic. High-level aminoglycoside-resistant (HLAR) enterococci are increasingly prevalent and preclude the use of this combination, posing a therapeutic challenge in persistent infections [1].

High-level aminoglycoside resistance is defined as a minimum inhibitory concentration (MIC) of >500 *µ*g/mL for gentamicin and an MIC of >1000 *µ*g/mL for streptomycin by broth diffusion [1]. The main mechanism of resistance involves the production of aminoglycoside-modifying enzymes, and additionally, mutations in a ribosomal protein for streptomycin [2].

The American Heart Association adult infective endocarditis guidelines currently recommend a combination of ampicillin plus ceftriaxone for HLAR *Enterococcus* infections, as evidence of synergy with this combination continues to grow [1]. The pediatric data for this combination, however, are scarce. Furthermore, ceftriaxone is not typically used in newborns due to risk of biliary sludging and precipitation with calcium-containing intravenous fluids such as total parenteral nutrition. At many institutions including ours, ceftriaxone is not recommended for prolonged use in infants less than 90 days corrected age. Thus, the optimal treatment of a newborn with HLAR *Enterococcus* endovascular infection is not clear.

We report a case of a neonate with persistent positive blood cultures for HLAR *Enterococcus faecalis* treated successfully with a combination of ampicillin and cefotaxime. Informed consent was obtained from the patient's family for this case report.

## 2. Case

A male infant was born at 26 4/7 weeks of gestation to a 30-year-old G3P2 mother who was blood group A+, HIV-negative, hepatitis B surface antigen-negative, rubella immune, and group B *Streptococcus*-negative. The mother's medical history was significant for cardiomyopathy that resulted in severe congestive heart failure during the pregnancy. This, in combination with fetal intrauterine growth restriction, necessitated induction of labor. The patient was born via vaginal delivery shortly after rupture of membranes. He required resuscitation with positive pressure ventilation and was intubated with subsequent stabilization. Apgar scores were 5 and 7 at 1 and 5 minutes of life, respectively. Birth weight was 490 g (<3rd percentile). He received ampicillin and tobramycin at birth, which were discontinued at 48 hours of life with a negative blood culture.

His postnatal course was complicated by respiratory distress syndrome requiring high-frequency oscillatory ventilation, pulmonary hemorrhage, anemia, thrombocytopenia, patent ductus arteriosus with bidirectional shunt, and grade II intraventricular hemorrhage. He was relatively stable until day of life (DOL) 9 when he developed abdominal distension and lethargy. His only intravenous (IV) access at this time was an umbilical venous catheter inserted shortly after birth. A full sepsis evaluation was performed, and treatment with IV vancomycin and tobramycin was commenced. White blood cell (WBC) counts increased from 19.5 to 30.3 × 10^9^/L, and C-reactive protein (CRP) increased from 18 to 69.7 mg/L over the course of one day. Peripheral blood, cerebrospinal fluid (CSF), and urine catheter cultures were all positive for *Enterococcus faecalis*. The organism was susceptible to ampicillin and vancomycin but was high-level aminoglycoside-resistant. Therefore, on DOL 11 when the blood culture sensitivities were known, vancomycin and tobramycin were discontinued, and ampicillin was started.

Peripheral blood cultures drawn on DOL 12 remained positive for *E. faecalis*, so vancomycin was added again on DOL 13 with the expectation of bloodstream clearance with two agents in a critically ill child. Despite treatment on ampicillin and vancomycin (with an acceptable vancomycin trough level of 8.8 mg/L), peripheral blood cultures continued to be positive on DOL 15 and 17. An umbilical arterial catheter had been removed on DOL 3, and the umbilical venous catheter was removed on DOL 11. No additional central access was placed while blood cultures remained positive. The patient received medications through a peripheral IV line. Investigations to identify a source of persistent infection, including an echocardiogram, abdominal ultrasound, and ultrasound of large vessels, were all unremarkable. Given the persistence of positive blood cultures from DOL 9 to 17, cefotaxime was added to ampicillin on DOL 17 for synergistic effect, and vancomycin was discontinued. A peripheral blood culture drawn on DOL 20 was finally negative.

As no endovascular source was confirmed, it was decided that the patient would complete a 2-week course with ampicillin and cefotaxime from the date of first negative culture. He did not experience any adverse effects to this antibiotic combination. He did not have any recurrence of *E. faecalis* infection but ultimately succumbed to multiorgan failure from his complicated postnatal course and died on DOL 70.

## 3. Discussion

While enterococci are intrinsically resistant to cephalosporins, there is increasing evidence that the combination of an aminopenicillin and third-generation cephalosporin (primarily cefotaxime or ceftriaxone) has synergistic activity against these organisms. Mainardi et al. demonstrated that amoxicillin and cefotaxime given together against *E. faecalis in vitro* lowered the MIC of each antibiotic significantly, regardless of the *E. faecalis* strain's susceptibility to aminoglycosides [3]. The proposed mechanism was a differential saturation of the penicillin binding proteins (PBPs), with cefotaxime saturating PBPs 2 and 3 and amoxicillin saturating PBPs 4 and 5 [3].

*In vivo* studies have since supported the effectiveness of the combination of ampicillin and ceftriaxone. For example, in 2007, Gavalda et al. compared 21 patients with HLAR *E. faecalis* endocarditis (age range 6 months to 82 years; median 65 years) with 22 patients with non-HLAR *E. faecalis* (age range 24 to 86 years; median 68 years) who were all treated with ampicillin and ceftriaxone. Clinical cure rates were similar in the two groups, with a 100% clinical and microbiological cure rate in the HLAR *E. faecalis* group who adhered to the study intervention [4].

Pediatric data regarding HLAR enterococci are currently limited to epidemiological studies assessing resistance patterns of blood and urine cultures. To our knowledge, there is only one published report of a pediatric patient in the literature who received the combination of ampicillin and third-generation cephalosporin for a HLAR *E. faecalis* endovascular infection. This patient was a 6-month-old infant who was part of the study by Gavalda et al. assessing ampicillin and ceftriaxone for HLAR *E. faecalis* infective endocarditis; this child demonstrated clinical and microbiologic cure [4]. In general, ceftriaxone is not used in the neonatal population and, instead, cefotaxime is used when a third-generation cephalosporin is warranted. Therefore, our case provides support for the use of the less-studied combination of ampicillin and cefotaxime in treating persistent infection with HLAR *E. faecalis.*

While we cannot be certain that the addition of cefotaxime to ampicillin in our patient was responsible for clearing his HLAR *E. faecalis* infection, we anticipate that the synergistic effect observed in the adult population may also be applied to the pediatric population. Another possible explanation for the persistent bacteremia in our patient may be a theoretical antagonistic effect between ampicillin and vancomycin; this phenomenon is inconsistent in the literature, however, with uncertain clinical significance [5].

To our knowledge, this is the first case illustrating the use of ampicillin and cefotaxime for a HLAR *E. faecalis* infection in a neonate. Further research is required in the pediatric population to assess the efficacy and safety of ampicillin and cefotaxime/ceftriaxone in the treatment of persistent infections with increasingly prevalent HLAR *Enterococcus*.
